# Myeloid-Derived Suppressor Cell-Derived Arginase-1 Oppositely Modulates IL-17A and IL-17F Through the ESR/STAT3 Pathway During Colitis in Mice

**DOI:** 10.3389/fimmu.2020.00687

**Published:** 2020-04-22

**Authors:** Zhanchuan Ma, Yu Zhen, Cong Hu, Huanfa Yi

**Affiliations:** ^1^Central Laboratory, The First Hospital of Jilin University, Changchun, China; ^2^Key Laboratory of Organ Regeneration and Transplantation, Ministry of Education, Changchun, China; ^3^Department of Dermatology, The First Hospital of Jilin University, Changchun, China

**Keywords:** myeloid-derived suppressor cells, arginase-1, TH17 cell, IL-17A, IL-17F

## Abstract

Myeloid-derived suppressor cells (MDSC) play a crucial role in regulating the intestinal immune response during colitis. We previously revealed an essential role of MDSC in promoting TH17 cell polarization, which was found to be arginase-1 (Arg-1)-dependent; however, the underlying mechanism remains obscure. Here we report that percentage of MDSC decreased in Arg^*myeKO*^ mice during DSS-induced colitis. IL-17A levels reduced but IL-17F levels increased significantly in the colorectum of Arg^*myeKO*^ mice, leading to severe tissue damage and high risk of mortality rate. Activation of estrogen receptor (ESR) increased pSTAT3 level in MDSC and consequently led to elevated percentage of MDSC and more Arg-1 and inducible nitric oxide synthase expression in MDSC. Increased level of IL-17A and reduced level of IL-17F alleviated colitis in mice consequently. Together, these findings demonstrate a protective role of MDSC-derived Arg-1 during colitis after activates ESR/STAT3 signaling in MDSC. High level of Arg-1 favors accumulation of IL-17A, but reduced IL-17F expression in the colorectum of mice and ultimately leading to relief of colitis, indicating a potential clinical impact of MDSC-derived Arg-1 for controlling inflammatory bowel disease.

## Graphical Abstract

Schematic diagrams for the mechanisms of MDSC-derived Arg-1 in the polarization of TH17 cells in DSS-induced colitis mouse model. Quercetin binds to ESR expressed by MDSC, activates STAT3 and promotes Arg-1 secretion in MDSC. High level of Arg-1 facilitates TH17 cell polarization and enhances IL-17A expression but inhibits IL-17F level in the colorectum during colitis.

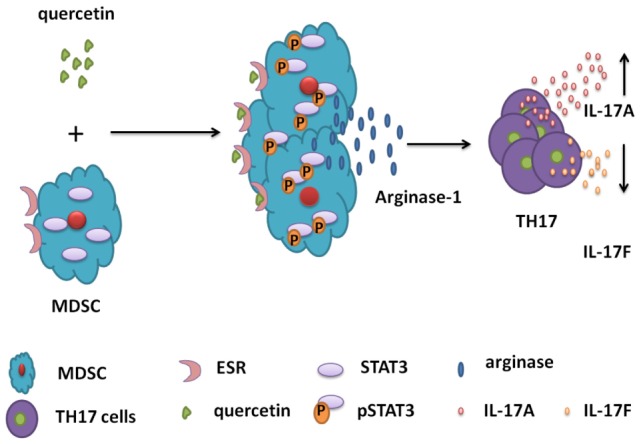

## Introduction

Inflammatory bowel disease (IBD) is a chronic and relapsing disorder that involves the whole intestinal system, and generally indicates ulcerative colitis (UC) and Crohn’s Disease (CD) in human. IBD causes diarrhea, mulligrubs, fever, hematochezia and weight loss, and occurs globally. IBD gives rise to colorectal carcinoma and shortens the lifespan of patients and as such, can be fatal (1, 2). When the epithelial barrier is broken, microbial antigens and self-antigens are processed and presented by antigen-presenting cells, which elicit fierce pro-inflammatory activity after activation, recruiting various immune cells to the inflammatory sites and attacking intestinal cells (3–5).

Both innate and adaptive immune cells participate in IBD progression by secreting various cytokines (6). TH17 cells are critical in the regulation of the immune response in IBD progression (7), which are the primary source of IL-17A and IL-17F (8). Generally, there are two IL-17A receptors: IL-17RA and IL-17RC. IL-17F is closely related to IL-17A, and these two cytokines bind to IL-17RA or IL-17RC as homodimers (IL-17A-IL-17A, IL-17F-IL-17F) or heterodimers (IL-17A-IL17F) to activate downstream signals (8, 9). IL-17F-/- mice showed a higher resistance to IBD induction as compared with IL-17A knockout mice (10), and neutralization of IL-17F, not IL-17A, alleviated colitis progression in mice (11), indicating a pathologic role of IL-17F in autoimmune disease. In patients with psoriatic arthritis, bimekizumab treatment neutralizes IL-17A and IL-17F greatly inhibits pro-inflammatory cytokine secretion, but IL-17A blockade in IBD patients induced high risk of severe adverse events in clinical trials (12, 13), indicating a protective role of IL-17A in IBD.

MDSC is a cluster of heterogeneous cells that are consisting of immature monocytes, neutrophils and dendritic cells (DCs), which are derived from the bone marrow and co-expression of CD11b and Gr-1 in mice (14, 15). Generally, MDSC can be divided into two subsets base on different markers: CD11b^+^Gr-1^+^Ly 6G^+^ Ly 6C^*low*^ granulocytic MDSC (G-MDSC) and CD11b^+^Gr-1^+^Ly 6C^*high*^ Ly 6G^–^ monocytic MDSC (M-MDSC) (16). Meanwhile, there are different strategies to define subpopulations of MDSC: G-MDSC was defined as CD11b^+^Ly6G^+^Ly6C^*low*^, and M-MDSC was defined as CD11b^+^Ly6C^*high*^Ly6G^–^(17). Trauma, infection, cancer and abnormal inflammatory conditions promote MDSC proliferation, and MDSCs are recruited to the inflammatory microenvironment, exerting immunosuppressive activities by secreting Arg-1, inducible nitric oxide synthase (iNOS) and indolamine dioxygenase (IDO), which inhibit TCR ζ-chain synthesis and promote T-cell apoptosis (18). Clinical studies have revealed that the MDSC fraction correlates with disease severity in ulcerative colitis or Crohn’s disease (19). Adoptive transfer of MDSCs in IBD mice led to Treg expansion, disease symptom alleviation, and suppression of secretion of cytokines such as IL-17A and TNF-α (20). MDSC elimination resulted in aggravation of colitis symptoms (20). Our previous studies revealed an essential role of MDSC in promoting TH17 cell polarization, which was found to be Arg-1-dependent. We demonstrated that Arg-1 and IL-1β secreted by MDSC drive TH17 cell differentiation in mouse models and patients with systemic lupus erythematosus (SLE) and arthritis (14, 21).

Arg-1 is an ureohydrolase produced predominately in liver, but also be secreted by MDSC (22). It plays a crucial role in the urea cycle (23). Both Arg-1 and iNOS modulate the immune response by catalyzing L-arginine, an amino acid that is indispensable for T-cell CD3ζ-chain synthesis (24). A previous study in a mouse model demonstrated that Arg-1 deficiency aggravates contact hypersensitivity due to excessive NO accumulation (25). Intraperitoneal injection of the Arg-1 inhibitor nor-NOHA effectively alleviated mouse colitis progression (26).

In our studies, Arg^*myeKO*^ mice with dextran sodium sulfate (DSS)-induced IBD were used to identify the role of MDSC and IL-17 in colitis. We found that Arg^*myeKO*^ mice suffered severe IBD than did WT mice because of decreased percentage of MDSC and reduced IL-17A level under IBD conditions. Whereas IL-17F level was increased, leading to colitis progression in Arg^*myeKO*^ mice. Quercetin treatment activated ESR signal in MDSC and in turn boosted STAT3 phosphorylation, dramatically increased MDSC percentage and up-regulated Arg-1 and iNOS in MDSC during colitis. Elevated Arg-1 enhanced IL-17A expression but decreased IL-17F levels and contributed to attenuating immune response in the colorectum, thereby maintained intestinal barrier integrity and ultimately alleviating colitis.

## Materials and Methods

### Animals

Female 6–8 weeks old mouse maintained under specific pathogen-free (SPF) conditions were used to perform animal experiment. C57BL/6 mice and Arg^*myeKO*^ mice were used to construct IBD model. Arg^*flox/flox*^ mice crossed to the lyz2-Cre mice to generate Arg^*myeKO*^ mice. Arg^*myeKO*^ mice with deficiency of Arg-1 in myeloid cells were confirmed with PCR and western blot. All mice were obtained from the Jackson Laboratory (Bar Harbor, ME, United States). All animal experiments were approved by the Subcommittee on Research Animal Care of the First Hospital of Jilin University.

### IBD Induction

40–50 KDa DSS (Sigma-Aldrich) was dissolved at final concentration of 3.5% in water and give to mice orally for 9 days or more days to establish an enteritis model. Weight loss, stool consistency and rectal bleeding were monitored daily to evaluate the disease activity index (DAI) of enteritis in mice. DAI scores were recorded as 0–3 based on severity of illness. Stool consistency: 0 = normal, 1 = soft but still formed, 2 = very soft, 3 = diarrhea. Rectal bleeding: 0 = negative hemoccult, 1 = positive hemoccult, 2 = blood traces in stool visible, 3 = rectal bleeding. The DAI was calculated as the mean value of the combined scores of stool consistency and rectal bleeding of each mouse.

### Drug Treatment

Quercetin (Shanghai Aladdin) was dissolved in dimethylsulfoxide upon receipt. Stock solution (1mmol/L) was stored as aliquots at –80°C under sterile conditions. Mice were treated with quercetin (0.5 μM/g, diluted with PBS) was described previously (27). Briefly, mice were intraperitoneally injected with quercetin or vehicle (300μl per mouse) at day 0, 3, 5 and 7. From day 0, mice were treated with 3.5% DSS for 8–10 days, then sacrificed for further studies.

### Cell Isolation

Single cell suspensions of peripheral blood mononuclear cells (PBMC) and spleen cells were generated as described previously (21). Peyer’s patches (PP) were carefully detached from the intestine to collect immune cells. To collect lamina propria mononuclear cells (LPMC), the colon was cut into small pieces and washed in phosphate-buffered saline (PBS), and the pieces were shaken in 50 ml of buffer (PBS containing 5 mM EDTA and penicillin/streptomycin) at 37°C for 30 min to remove intraepithelial lymphocytes. Then, the colon pieces were cut into 1-mm^3^ pieces, digested with 1 mg/ml collagenase IV and 10 U/ml DNase I at 37°C for 1 h, and filtered through 70-μm cell strainers to obtain LPMC. The cells were then centrifuged in a Ficoll-hypaque gradient for 30 min and LPMC were collected.

### Flow Cytometry

For flow cytometric analysis, cells were performed according to standard procedures. Before IL-17A and IL-17F detection, 2 × 10^5^ cells stimulated with cocktail as manufacturer’s specification (BD Biosciences). Single cell suspension was washed twice in FACS buffer and stained for CD3 pecy7 (17A2), CD4 percpcy5.5 (GK1.5), CD25 PE (3C7), γδT BV421 (GL3), CD11b apccy7 (M1/70), Gr-1 percpcy5.5 (RB6-8C5), Ly 6C pecy7 (AL-21), Ly 6G FITC (RB6-8C5), IL-17A APC (TC11-18H10.1), IL-17F PE (9D3.1C8), Foxp3 AF488 (150D), pSTAT3 PE (13A3-1), IL-22 PE (Poly5164) and Arg-1 PE (Polyclonal). Antibodies for γδT BV421, Ly 6C and Ly 6G were purchased from BD biosciences, antibody for Arg-1 was purchased from R&D systems, rest antibodies were purchased from Biolegend. Flow cytometric analyses were conducted using an Ariall flow cytometer (BD Biosciences). Data were evaluated using FlowJo software (Version 10; FlowJo).

### *In vitro* Suppression Assays

Before suppression assays, 96-well plates were incubated with 2 ug/ml anti mouse CD3 purified antibody overnight in 4°C conditions. Cells from lymph nodes of mice were stained with 2.5 μM of CFSE (eBioscience) for 15 min, and then stopped by the addition of complete RPMI 1640 medium (HyClone). After several washes, CFSE-labeled cells were incubated for 72 h in the presence of 1 ug/ml anti mouse CD28 purified antibody for 72 h.

To purify MDSC, CD11b + Gr-1 + cells were isolated from spleen of colitic mice according to the manufacturer’s instruction. Briefly, red blood cells were lysed and splenic cells were washed in FACS buffer and then incubated with CD11b apccy7, Gr-1 percpcy5.5 antibodies for 30 min at 4°C. Then CD11b^+^Gr-1^+^ cells were sorted (purity > 95%) and co-cultured with T cells for 72 h. Proliferation of T cells was determined based on CFSE dilution by flow cytometry.

### Adoptive Transfer

MDSC were isolated from spleen of colitic mice and suspended in PBS. 2 × 10^6^ WT-derived MDSC were transferred intravenously into colitic Arg^*myeKO*^ mice on days 0 and 2 after DSS administration. Mice were monitored daily for clinical signs of disease (28).

### Quantitative Reserve Transcription RT-qPCR

Total RNA was isolated from colonic tissues using TRIzol reagent (Invitrogen Life Technologies, Carlsbad, CA, United States) according to the manufacturer’s instructions. gDNA was removed and cDNA was synthesized following the manufacturer’s specifications (Yeasen, Shanghai, China). qPCR were run in a StepOnePlus Real-Time PCR System (Applied Biosystems, Carlsbad, CA, United States) using commercial kits (TransGen Biotech, Beijing, China) according to the manufacturer’s instructions. mRNA levels of target genes were normalized against the β-actin mRNA level and relative gene expression was determined using the 2^–Δ^ ^Δ^ ^*Ct*^ method. The primer sequences set used for Arg-1, iNOS, IDO, IL-17A, IL-22, IL-23, OCLN, ACT1 and β-actin were listed in Table 1.

**TABLE 1:** Primer sequences.

Primer name	Primer sequence
β-actin	F: 5′ TTCAACACCCCAGCCATG 3′
	R: 5′ CCTCGTAGATGGGCACAGT 3′
Arg-1	F:5′TGTCCCTAATGACAGCTCCTT 3′
	R:5′GCATCCACCCAAATGACACAT 3′
iNOS	F:5′TGGCCACCTTGTTCAGCTACG 3
	R: GCCAAGGCCAAACACAGCATAC 3′
Il-17A	F:5′ GGCCCTCAGACTACCTCAAC 3′
	R:5′ TCTCGACCCTGAAAGTGAAGG 3′
IL-17F	F:5′ TGCTACTGTTGATGTTGGGAC 3′
	R:5′ CAGAAATGCCCTGGTTTTGGT 3′
IL-22	F:5′ CGCTGCCCGTCAACACCCGG 3′
	R:5′ CTGATCTTTAGCACTGACTCCTCG 3′
IL-23	F:5′ GGACTCAGCCAACTCCTCC 3′
	R:5′ GCTCCGTGGGCAAAGAC 3′
COX-2	F:5′ TTCTCTACAACAACTCCATCCTC 3′
	R:5′ GCAGCCATTTCCTTCTCTCC 3′
OCLN	F:5′ TGAAAGTCCACCTCCTTACAGA 3′
	R:5′ CCGGATAAAAAGAGTACGCTGG 3′
ACT1	F:5′ TGCCTGCGAGCTAAAGTCC 3′
	R:5′ CCGTAAGTGTGAACCGATGCT 3′

### Histological Assessment

Distal colon tissues were washed in PBS to remove fecal matter. The tissue was fixed in 10% neutral phosphate-buffered formalin, embedded in paraffin, and stained with hematoxylin and eosin (H&E). Sections of fixed colon were imaged with the Zeiss Mirax Midi scanner.

### Statistical Analysis

Data are expressed as mean ± SD. Where indicated, statistical analysis was performed on Prism 7.0 (GraphPad Software). Student’s *t*-test and one-way ANOVA were used as apt to compare means. A *P*-value < 0.05 was considered significant. Statistical analysis was performed using Prism 7.0 (GraphPad Software).

## Results

### Arg^*myeKO*^ Mice Exhibit Severe IBD After DSS Treatment

**FIGURE 1:**
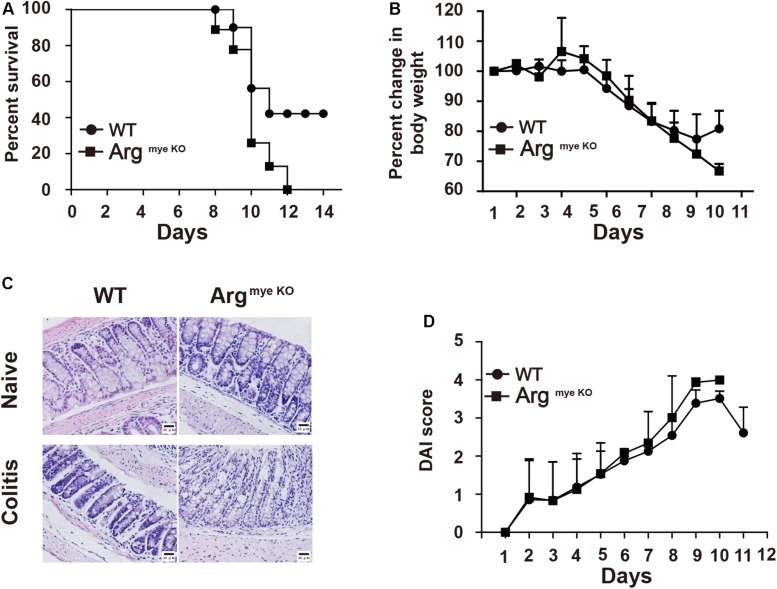
Arg^*myeKO*^ mice suffered severe colitis. WT and Arg^*myeKO*^ mice treated with 3.5% DSS for 11 days, survival **(A)**, body weight (relative to original weight, set as 100%) **(B)** and histology (H&E staining) **(C)** of mice was monitored every day. DAI score was calculated as indicated in Materials and Methods. Scale bar = 20 μm. (WT, *n* = 5; Arg^*myeKO*^, *n* = 7). Data are representative of three independent experiments with similar results. Quantification of signal was shown in bar graphs and error bars represent mean ± SD.

Arg-1 secreted from MDSCs reportedly plays an important role in IBD progression (29). However, the role of Arg-1 in IBD has not been examined directly. Here we treated Arg^*myeKO*^ and WT mice with 3.5% DSS for 11 days and survival curve of mice were monitored. All Arg^*myeKO*^ mice died within 12 days, whereas 40% of the WT mice were still alive at this time point (Figure 1A). Body weight decreased rapidly after DSS treatment in both treatment groups; however, Arg^*myeKO*^ mice exhibited a more dramatic weight loss as compared with that of WT mice (Figure 1B). H&E staining of colorectal tissues revealed that more immune cells had infiltrated the colorectums of Arg^*myeKO*^ mice after DSS treatment than those in the WT group. More goblet cells depletion was also observed in the colorectum of Arg^*myeKO*^ mice (Figure 1C). The DAI showed that Arg^*myeKO*^ mice obtained higher scores than did WT mice, reflecting more serious pathologic changes in Arg^*myeKO*^ mice (Figure 1D). Together, these results indicated a central role of MDSC-derived Arg-1 in alleviating immune response during acute colitis.

### Increased IL-17F and Reduced IL-17A Levels in the Colorectum Aggravate Colitis in Arg^*myeKO*^ Mice

**FIGURE 2:**
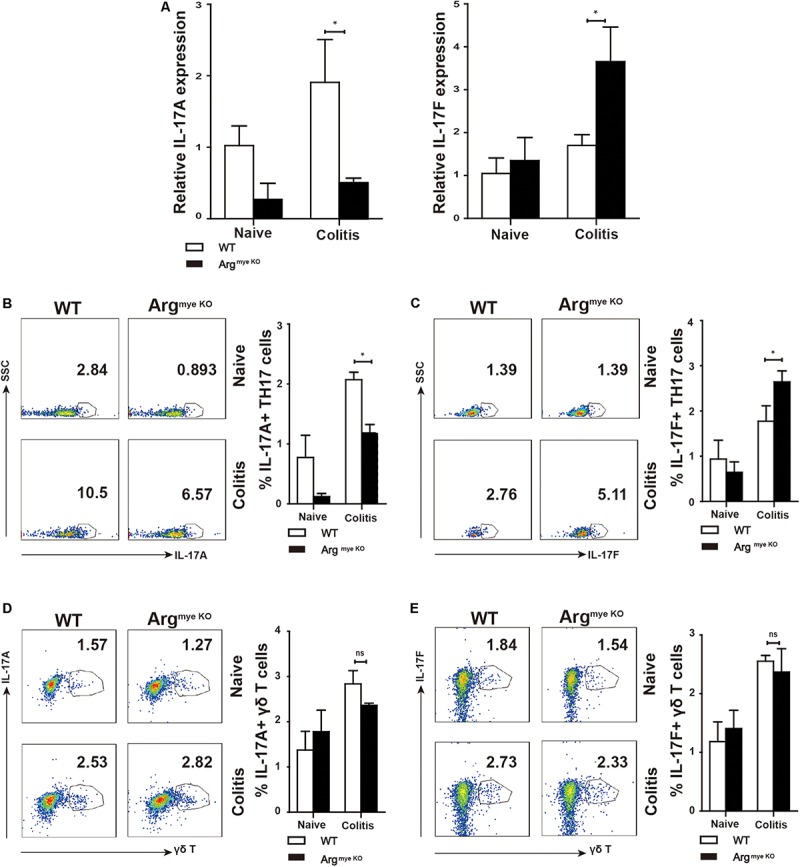
Distinct roles of IL-17F and IL-17A in regulating intestinal immune response during colitis. WT and Arg^*myeKO*^ mice treated with 3.5% DSS for 9 days. Mice were sacrificed and the colorectum was separated to isolate mRNA for further studies. PP was isolated to generate signal cell suspension. Before IL-17A/F detection, cells were stimulated with cocktail for 6 h. *P < 0.05; **P < 0.01. Data are representative of three independent experiments with similar results. Quantification of signal was shown in bar graphs and error bars represent mean ± SD. **(A)** qPCR analysis of IL-17A and IL-17F in the colorectum of WT and arg^*myeKO*^ mouse. **(B,C)** Flow cytometry of TH17 cells in PP from WT and arg^*myeKO*^ mice, gated on CD4 + T cells. **(D,E)** Flow cytometry of γδT cells in PP from WT and arg^*myeKO*^ mice, gated on CD3 + T cells.

IL-17A and IL-17F play critical roles in the intestinal immune response during colitis (8). IL-17A upregulation in the colorectum reportedly facilitated mucosal repair and gut epithelial integrity in a DSS-induced colitis model (30). A clinical trial revealed that IL-17F promotes an inflammatory response in patients with psoriatic arthritis (31). To evaluate IL-17A and IL-17F expression in mice with colitis, colorectal tissues collected from DSS-treated mice were subjected to RT-qPCR analysis. The results showed that IL-17A mRNA expression was reduced, whereas that of IL-17F was abnormally increased in the colorectums of Arg^*myeKO*^ mice (Figure 2A), indicating an opposite functions of IL-17A and IL-17F during colitis. IL-17A and IL-17F are secreted mainly by TH17 cells and γδT cells, which play a crucial role in the intestinal immune response (32). We detected a decrease in IL-17A expression in the PP in Arg^*myeKO*^ mice (Figure 2B). Intriguingly, IL-17F expression in the PP was higher than that in the WT group (Figure 2C). However, there was no significant difference in IL-17A and IL-17F expression in γδT cells between the two groups (Figures 2D,E). These data suggested a protective role of IL-17A and a pathogenic effect of IL-17F in the intestinal immune response during colitis.

### Suppressive Factors Are Down-Regulated in Arg^*myeKO*^ MDSC During Colitis

**FIGURE 3:**
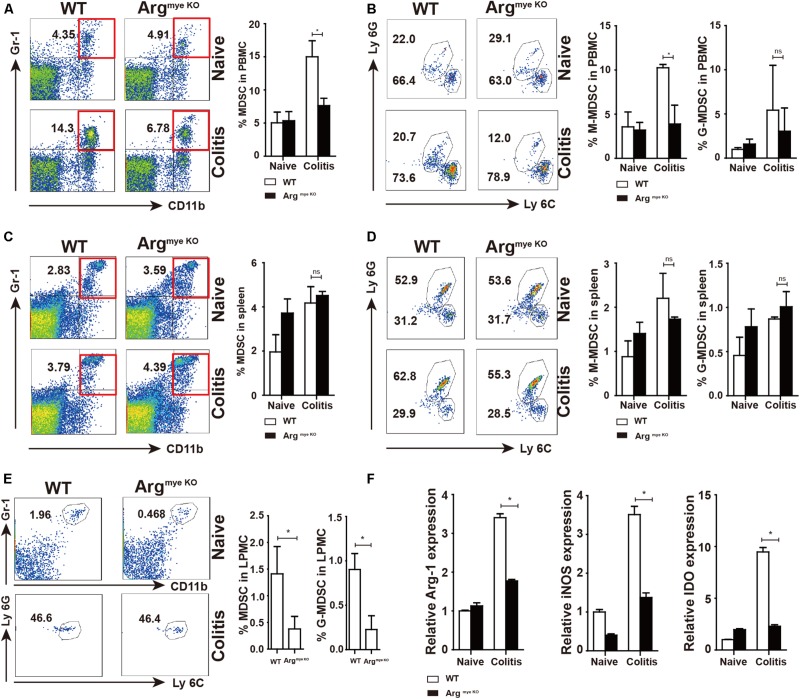
Decreased percentages and impaired suppressive factors level in MDSC of arg^*myeKO*^ mouse during colitis. WT and Arg^*myeKO*^ mice treated with 3.5% DSS for 9 days. Mice were sacrificed and PBMC, splenic cells and LPMC were isolated to detect MDSC as indicated. Flow cytometry of MDSC showing the MDSC in PBMC **(A,B)**, spleen **(C,D)**, LPMC **(E)**, **(F)** qPCR analysis of Arg-1, iNOS and IDO in the colorectum of WT and arg^*myeKO*^ mice. *P < 0.05. Data are representative of three independent experiments with similar results. Quantification of signal was shown in bar graphs and error bars represent mean ± SD.

Our results showed percentage of MDSC increased in PBMC of colitis mice when compared to that in control mice (Figure 3A). However, the increase in the MDSC population was smaller in the Arg^*myeKO*^ than in the WT mice. M-MDSC (CD11b^+^Gr-1^+^Ly6C^+^) were the predominant subpopulation of MDSC, which was smaller in Arg^*myeKO*^ mice than in the WT group, whereas Arg-1 deficiency did not impact G-MDSC (CD11b^+^Gr-1^+^Ly6G^+^) in PBMC (Figure 3B). Splenic MDSC levels were not significantly different between the two groups (Figures 3C,D). The MDSC fraction in LPMCs of Arg^*myeKO*^ mice was also significantly decreased after DSS treatment (Figure 3E). Further, the expression of Arg-1, iNOS and IDO, which are crucial for the suppressive function of MDSC, was decreased in the colorectums of Arg^*myeKO*^ mice (Figure 3F), leading to impaired suppressive function of Arg^*myeKO*^-derived MDSC (Supplementary Figure S2). It should be noted that Arg-1 gene was deleted specifically in myeloid cells, thus, the low intestinal Arg-1 expression observed might be due to endothelial cells, which also produce Arg-1 (33). Several articles have described the role of Tregs in IBD model mice (34, 35). However, we found no significant differences in Tregs in PBMCs, splenic cells, and PP cells between Arg^*myeKO*^ and control mice (Supplementary Figure S1). Collectively, these findings revealed that Arg-1 deletion resulted in decreases in the MDSC population and MDSC suppressive factors expression during acute colitis.

### Arg-1 Deletion Results in the Suppression of IL-17A-Downstream Gene Expression in the Colorectum

**FIGURE 4:**
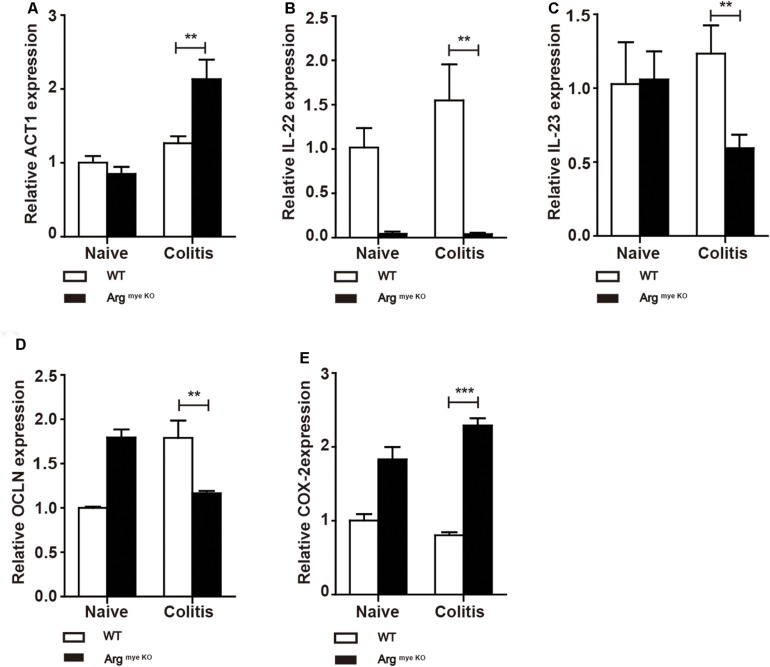
Protective factors decreased in the colorectum of arg^*myeKO*^ mouse during colitis. WT and Arg^*myeKO*^ mice treated with 3.5% DSS for 9 days. Mice were sacrificed and mRNA in the colorectum of mice was isolated for further studies. qPCR analysis of ACT1 **(A)**, IL-22 **(B)**, IL-23 **(C)**, OCLN **(D)**, and COX-2 **(E)** in the colorectum of WT and arg^*myeKO*^ mice. **P < 0.01; ***P < 0.001. Data are representative of three independent experiments with similar results. Quantification of signal was shown in bar graphs and error bars represent mean ± SD.

IL-17A has a crucial role in intestinal immunity, autoimmune disease and host defense (9). IL-17A is generally associated with IL-17F and through heterodimer formation, activates IL-17RA/IL-17RC signaling, which induces the secretion of a series of cytokines to elicit an intestinal immune response through ACT1 (9, 36). Indeed, ACT1 expression was elevated to various degrees in colitis mice (Figure 4A), whereas IL-22 level was decreased upon arg-1 deletion during colitis (Figure 4B and Supplementary Figure S3). The expression of IL-23, a key cytokine regulating TH17 cell proliferation and cytokine production (37), was decreased in Arg^*myeKO*^ mice (Figure 4C). In addition, occludin (OCLN), which plays a critical in maintaining intestinal barrier function, was decreased in colorectal tissues of Arg^*myeKO*^ mice during colitis (Figure 4D). Cyclooxygenase-2 (COX-2) was up-regulated in Arg^*myeKO*^ mice promoted colitis progression (Figure 4E). Collectively, these results demonstrated an attenuated protection in the intestines of Arg^*myeKO*^ mice due to decreased IL-17A level during colitis.

### WT-Derived MDSC Alleviated Colitis in Arg^*myeKO*^ Mice

**FIGURE 5:**
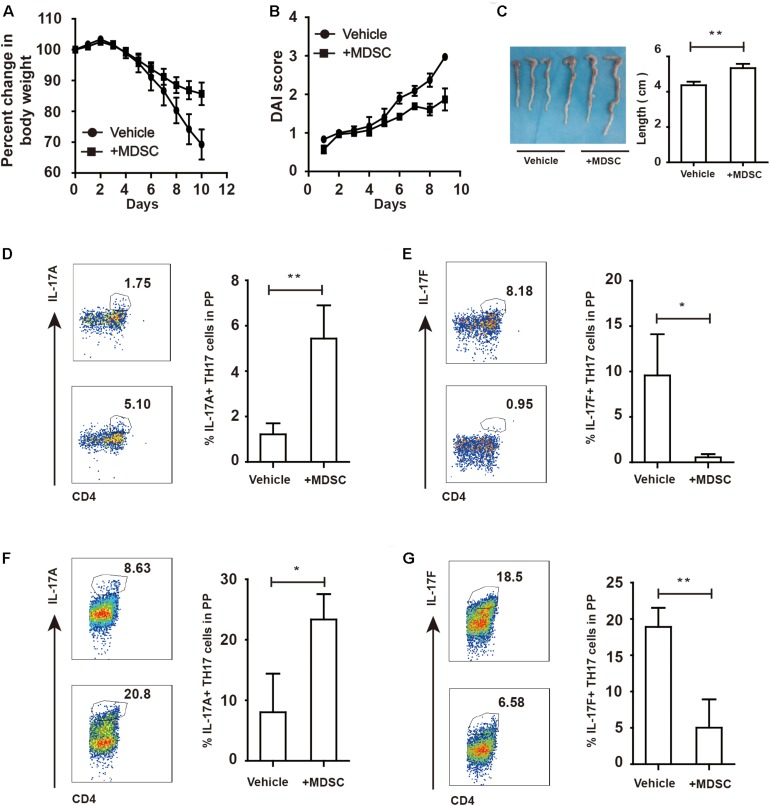
WT-derived MDSC alleviated colitis in arg^*myeKO*^ mice via regulating IL-17A and IL-17F in TH17 cells. Arg^*myeKO*^ mice treated with 3.5% DSS for 8 days and monitored every day, and 2 × 10^6^ WT-derived MDSC were transferred on day 0 and day 2. Then body weight **(A)**, DAI **(B)**, and colon length **(C)** was recorded (vehicle, *n* = 7; Arg^*myeKO*^, *n* = 7). Flow cytometry of TH17 cells in PP **(D,E)** and mLN **(F,G)** of arg^*myeKO*^ mice. *P < 0.05; **P < 0.01. Data are representative of three independent experiments with similar results. Quantification of signal was shown in bar graphs and error bars represent mean ± SD.

To investigate whether WT-derived MDSC could influence colitis progression in Arg^*myeKO*^ mice, we intravenously transferred WT-derived MDSC to Arg^*myeKO*^ mice during colitis. The results showed that transfer of MDSC reversed weight loss of colitic Arg^*myeKO*^ mice (Figures 5A,B). And colitic mice without transfer of MDSC had shorter colorectum in length (Figure 5C). In addition, transfer of MDSC significantly elevated IL17A level in TH17 cells in PP and mLN of colitic Arg^*myeKO*^ mice (Figures 5D,F), while IL-17F level was reduced and eventually prolonged survival of colitic Arg^*myeKO*^ mice (Figures 5E,G), indicating a key role of MDSC-derived Arg-1 in regulating TH17 cells and controlling colitis progression in Arg^*myeKO*^.

### Expanded MDSC Alleviate DSS-Induced Colitis

**FIGURE 6:**
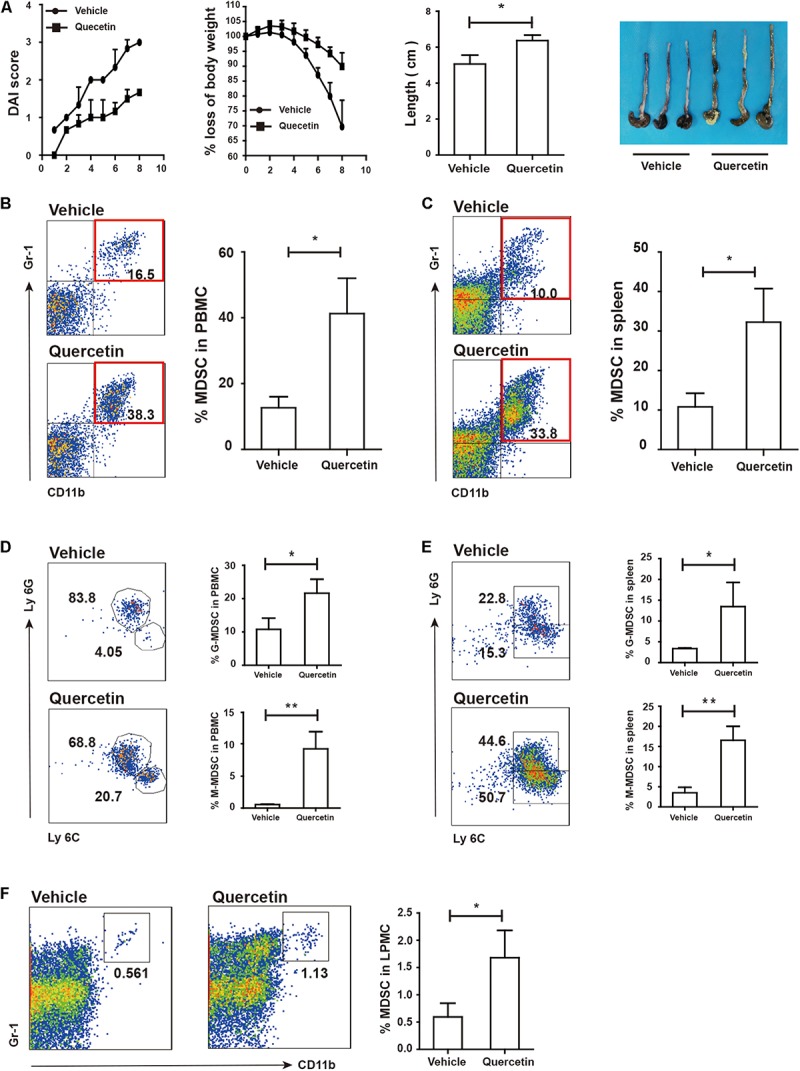
Quercetin treatment alleviated colitis depended on MDSC expansion. WT mice treated with 3.5% DSS for 8 days and monitored every day, then DAI, body weight and colon length **(A)** was recorded (WT, *n* = 3; Arg^*myeKO*^, *n* = 3). Flow cytometry of MDSC in PBMC **(B)**, spleen **(C)**, and LPMC **(F)** of WT and arg^*myeKO*^ mice. Subpopulation of MDSC in PBMC **(D)** and spleen **(E)** of WT and arg^*myeKO*^ mice. *P < 0.05; **P < 0.01. Data are representative of three independent experiments with similar results. Quantification of signal was shown in bar graphs and error bars represent mean ± SD.

Quercetin is a natural product ubiquitously exists in leaves, fruits, seeds of plants (38). Quercetin treatment ameliorated DSS-induced colitis (39); however, its contribution to MDSC and TH17 cells remained unclear. We found that quercetin treatment significantly alleviated colitis in mice, as evidenced by lower DAI scores, reduced body weight loss, and longer colon length (Figure 6A). The MDSC population in PBMC and spleen of colitis mice was highly increased after quercetin treatment (Figures 6B,C). G-MDSC and M-MDSC populations were also significantly expanded upon quercetin treatment (Figures 6D,E). Finally, MDSC levels in LPMC were also increased after quercetin treatment (Figure 6F). Collectively, these data revealed that quercetin treatment improved colitis by promoting MDSC expansion.

### Activated ESR/STAT3 Elevate Arg-1 Level in MDSC

**FIGURE 7:**
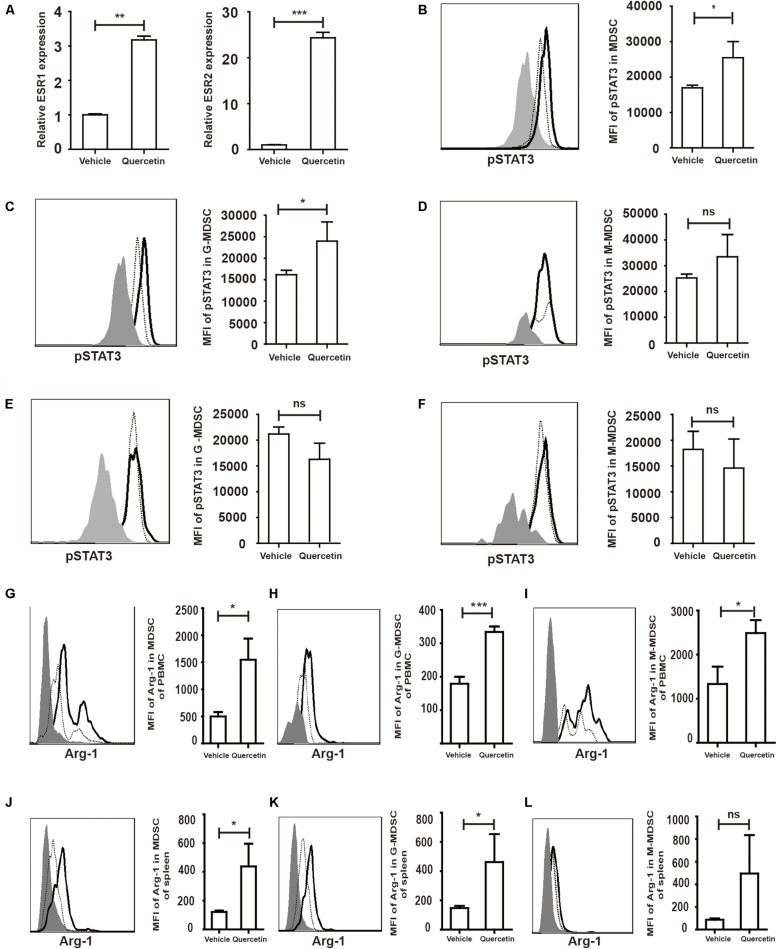
Quercetin enhanced suppressive factors expression in MDSC by improved pSTAT3 levels after binding to ESR. WT mice treated with 3.5% DSS for 9 days. Mice were sacrificed and qPCR analysis of ESR1, ESR2 **(A)**, Arg-1 and iNOS **(G)** of the colorectum in WT and arg^*myeKO*^ mouse during colitis. Flow cytometry of pSTAT3 of MDSC in PBMC **(B–D)** and spleen **(E,F)**. Flow cytometry of Arg-1 in MDSC **(G)** and subpopulations **(H,I)** of PBMC; Flow cytometry of Arg-1 in MDSC **(J)** and subpopulations **(K,L)** of spleen, gray, isotype; dotted, vehicle treatment; solid, quercetin treatment. *P < 0.05; **P < 0.01; ***P < 0.001. Data are representative of three independent experiments with similar results. Quantification of signal was shown in bar graphs and error bars represent mean ± SD.

Quercetin stimulates cell proliferation after binding to the estrogen receptor (ESR) (40). Estrogen drives the accumulation of MDSC and enhances the suppressive function of MDSC by activating STAT3 (41). We hypothesized that MDSC were stimulated by quercetin via binding of the latter to the ESR. Increased expression of both ESR1 and ESR2 in the colorectum suggested that ESR expressed by MDSC may recognize and bind to quercetin (Figure 7A). STAT3 has multiple biological activities involved in MDSC survival, proliferation, differentiation and apoptosis (42). Flow-cytometric analysis revealed that pSTAT3 expressed in MDSC from PBMC was increased upon quercetin treatment (Figure 7B and Supplementary Figure S4). G-MDSC, but not M-MDSC, more strongly expressed pSTAT3 in PBMC upon quercetin treatment (Figures 7C,D). In splenic MDSC, pSTAT3 expression was not affected by quercetin stimulation (Figures 7E,F). Activation of STAT3 enhanced Arg-1 synthesis in MDSC (Figure 7G), and subpopulations of MDSC also secreted more Arg-1 in PBMC after quercetin treatment (Figures 7H,I). In addition, quercetin also induced elevated Arg-1 secretion in splenic MDSC (Figure 7J), mainly G-MDSC (Figures 7K,L). Collectively, these experiments demonstrated a key role of ESR/STAT3 in enhancing the suppressive factors secretion in MDSC after quercetin treatment.

### High Level of Arg-1 in MDSC Promoted TH17 Cell IL-17A Secretion by Inhibiting IL-17F Levels

**FIGURE 8:**
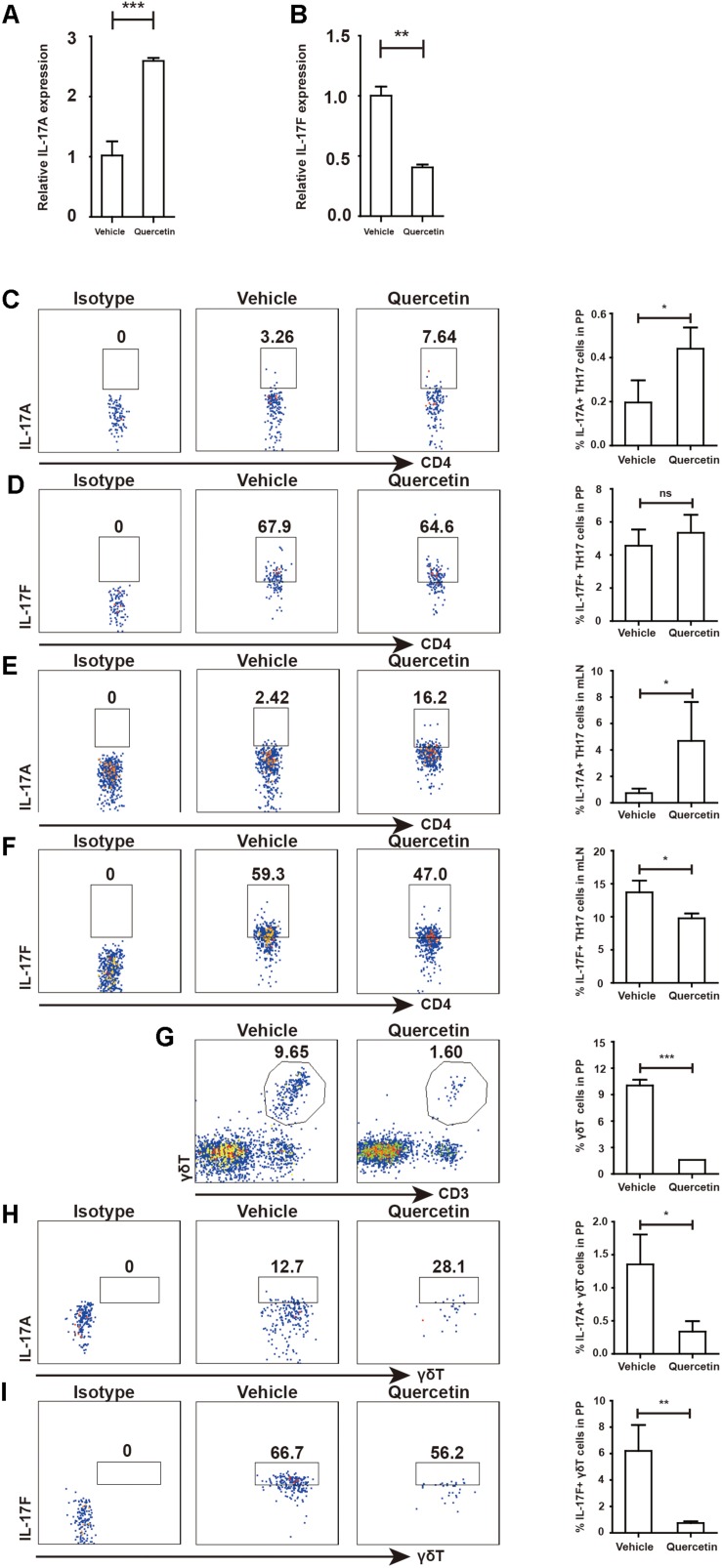
MDSC-derived Arg-1 enhanced IL-17A, but decreased IL-17F expression in TH17 cells WT mice treated with 3.5% DSS for 9 days. Mice were sacrificed and qPCR analysis of IL-17A **(A)** and IL-17F **(B)** in the colorectum of WT and arg^*myeKO*^ mouse during colitis. Before IL-17A/F detection, cells were stimulated with cocktail for 6 h. Flow cytometry of TH17 cells in PP **(C,D)** and mLN **(E,F)**. Flow cytometry of γδT cells in PP **(G–I)**. *P < 0.05; **P < 0.01; ***P < 0.001. Data are representative of three independent experiments with similar results. Quantification of signal was shown in bar graphs and error bars represent mean ± SD.

Our previously studies demonstrated that Arg-1 secreted by MDSC facilitates TH17 cell polarization in SLE patients and patients with arthritis (14, 21). To test whether quercetin induced MDSC-derived Arg-1 could promote TH17 cell responses, we measured TH17 cells in the PP and mesenteric lymph nodes (mLN) after quercetin treatment. IL-17A expression in the colorectum was elevated after quercetin treatment, whereas that of IL-17F was significantly reduced (Figures 8A,B). We observed significant increases in CD4^+^IL-17A^+^ TH17 cells in the PP and mLN respectively (Figures 8C,E). Intriguingly, IL-17F production by TH17 cells was decreased in the mLN, but not in the PP (Figures 8D,F), which is in line with a report that lower IL-17F level alleviates DSS-induced colitis in mice (11). γδT cells are another main source of IL-17A, we found that the population of γδT cells in the PP was reduced after quercetin treatment (Figure 8G). Expression of IL-17A and IL-17F was also reduced in γδT cells (Figures 8H–I). In addition, absolute numbers of IL-17A + TH17 cells also increased in PP and mLN of colitic mice after quercetin treatment (Supplementary Figure S5). Collectively, MDSC-derived Arg-1 promotes IL-17A expression in the colorectum, inhibits IL-17F expression and greatly relives colitis in mice.

### Protective Factors in the Colorectum Were Up-Regulated After Activating IL-17A Signal

Given the opposite roles of IL-17A and IL-17F in colitis, we next evaluated genes regulated by IL-17 A/F. After IL-17A/F is released by TH17 cells and γδT cells, these cytokines activate ACT1 by recognizing IL-17RA/RC upon homodimer or heterodimer formation (8). Indeed, ACT1 expression in the colorectum was increased during colitis (Figure 8A). Activation of ACT1 led to overexpression of IL-22, IL-23 and OCLN (Figures 8B–D and Supplementary Figure S6). Expression of the COX-2 in the colorectum during colitis was suppressed by quercetin treatment (Figure 8G). Collectively, attenuated colitis requires increased IL-17A in the colorectum, but not IL-17F, to initiate protective factor synthesis.

## Discussion

**FIGURE 9:**
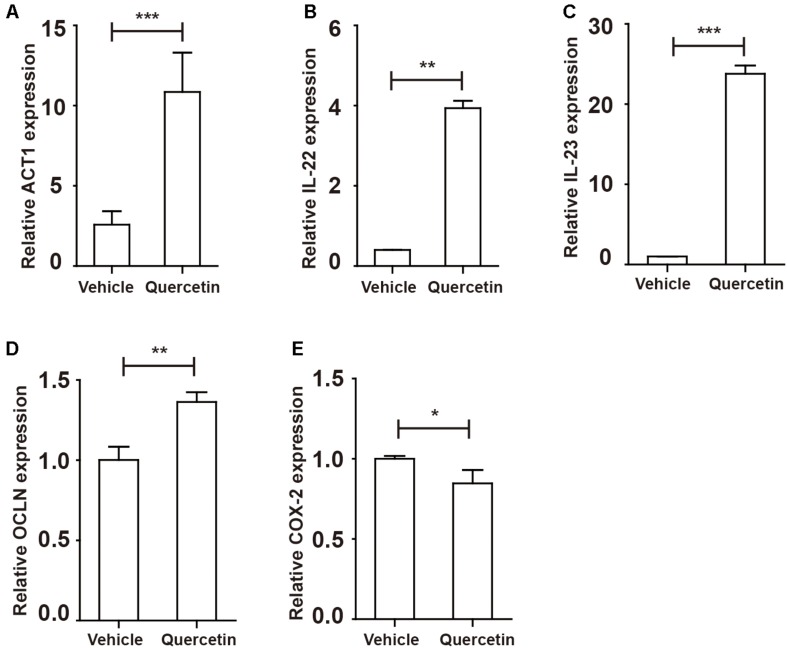
Protective factors were up-regulated in the colorectum of colitis mice after quercetin treatment. WT mice treated with 3.5% DSS for 9 days. Mice were sacrificed and qPCR analysis of ACT1 **(A)**, IL-22 **(B)**, IL-23 **(C)**, OCLN **(D)**, and COX-2 **(E)** in the colorectum of WT and arg^*myeKO*^ mice. *P < 0.05; **P < 0.01; ***P < 0.001. Data are representative of three independent experiments with similar results. Quantification of signal was shown in bar graphs and error bars represent mean ± SD.

In the present study, we demonstrated that Arg-1 deletion in myeloid cells promoted DSS-induced colitis progression via decreases in the MDSC and TH17 cell populations. Our results have shown that the absence of Arg-1 results in increased DAI score and poor prognosis in mice suffering DSS-induced colitis. Mice with a conditional deletion of Arg-1 in myeloid cells showed a decrease in percentage of MDSC in PBMC and LPMC. We have shown a decreased expression of IL-17A in the colorectum during colitis, while a significantly up-regulated IL-17F level adversely aggravated epithelial injury and hastened Arg^*myeKO*^ mice death eventually. The ESR1/2 activation up-regulated pSTAT3 level in MDSC, resulting in MDSC expansion and elevate suppressive factor secretion. Our data showed that high level of IL-17A production, often associated with TH17 cells in PP and mLN, obviously relieve weight loss of mice with colitis. We have identified distinct roles of IL-17A and IL-17F in modulating DSS-induced colitis in mice and we demonstrated the benefit of quercetin in colitis dependent on Arg-1 secreted by MDSC after ESR/STAT3 activation (Figure 9).

Our results supported a key role of MDSC-derived Arg-1 in regulating the immune response by inducing TH17 cell polarization in an IBD mice model. Our results showed that (1) a reduced frequency of MDSC in Arg^*myeKO*^ mice correlates with aggravated symptoms of IBD and (2) Arg-1 deletion impaired the suppressive factors secretion in MDSC thus feebly protecting mice from severe bleeding, weight loss and even death during colitis. Indeed, decreased Arg-1 aggravates DSS-induced colitis, presence of Arg-1 attenuate DSS-induced colitis (43, 44). In addition, we detected low expression of iNOS and IDO in Arg^*myeKO*^ mice, which were associated with suppressive function of MDSC. Mice with colitis and IBD patients who suffered ulcerative colitis and Crohn’s disease had an increased percentage of MDSC in PBMC during active and remission stages of diseases (19, 28). Adoptive transfer of MDSC successfully ameliorates colitis in mice models (20, 45, 46). The impaired MDSC thus failed to protect mice during colitis. We detected no changes of Tregs between WT and Arg^*myeKO*^ mice during colitis. One possible reason is that in approximately 8 days of acute colitis model, CD4^+^ T cells gradually entered a resting stage (47).

Although both IL-17A and IL-17F are produced by TH17 cells, we found lower expression of IL-17A, and significantly increased expression of IL-17F in Arg^*myeKO*^ mice during colitis. After binding to the IL-17RA/RC heteromeric receptor, IL-17A and IL-17F activates ACT1 to regulate immune responses in the colorectum (30, 48). We found that ACT1 expression was increased in the colorectum under physiological conditions. Activated ACT1 recruits TRAF6 and consequently induces NF-κB activation, resulting in an IL-17A signaling cascade (8). Our results showed that the expression of IL-17A target genes OCLN and IL-22 decreased in the colorectum of Arg^*myeKO*^ mice during colitis, which likely contributed to disruption of the intestinal barrier integrity thereby aggravating colitis. Intestinal IL-22 facilitates host antibacterial protein production and repair of the intestinal mucus barrier (49). Lack of IL-22 may account for insufficient intestinal mucus barrier repair and enhanced intestinal microfloral invasion during colitis, leading to the destruction of epithelial barrier integrity (30). Recent studies showed that IL-22 protected intestinal stem cells from carcinogenesis by initiating an intracellular DNA damage response (50). We also detected an increased IL-23 in the colorectum of Arg^*myeKO*^ mice during colitis, which was critical for IL-17A signal activation (30, 51). In the absence of IL-17A, increased tissue damage was observed in the colorectum of colitis mice. In addition, we detected overexpression of COX-2 after Arg-1 deletion, which promoted an inflammatory immune response in the colorectum (52). Accumulation of COX-2 in the colorectum inhibits intestinal epithelial regeneration, and COX-2–PGE2 pathway is crucial in regulating self-renewal and differentiation of intestinal epithelial stem cells during intestinal homeostasis (53). Together, these results suggested that Arg-1 deletion attenuates a protective role of MDSC during colitis by dichotomously regulating IL-17A and IL-17F levels in the colorectum, thus contributed to the exacerbation of colitis. Transfer of WT-derived MDSC alleviated colitis progression in Arg^*myeKO*^ mice evidenced by reversed weight loss of colitic Arg^*myeKO*^ mice, and IL-17A level significantly increased in PP and mLN of Arg^*myeKO*^ mice, indicating a key role of MDSC-derived Arg-1 in regulating TH17 cells and controlling colitis progression in Arg^*myeKO*^ mice. In addition, we observed that quercetin treatment attenuated colitis progression in Arg^*myeKO*^ mice to some extent (Supplementary Figures S7A,B), indicated that alternative pathway might participate in colitis therapy. One of the reasonable explanation is that the IBD is kind of autoimmune disease influenced by various factors (gut microbiota factor, immune factor, environmental factor and genetic factor) (54), pathogenesis of IBD still need further study; meanwhile as a natural product, quercetin has multiple biofunctions (38, 55). The interaction between quercetin and immune cells during colitis are still not fully well-explained. And further studies need to uncover the mechanisms involved in IBD.

The discovery of distinct functions of IL-17A and IL-17F produced by TH17 cells highlights a central role for this cell population in regulating adaptive immunity during colitis. Our previously study showed that Arg-1 secreted by MDSC facilitates TH17 cell polarization in SLE patients and patients with arthritis (14, 21). Our current study showed that quercetin treatment significantly attenuated weight loss and hematochezia in DSS-induced colitis mice by expanding the MDSC population. Proliferation and activation of MDSC can be induced by 17β-oestradiol via ESR and the STAT3-dependent pathway (41, 56). Quercetin effectively stimulated MDSC in DSS-induced colitis mice by activating ESR/STAT3, accompanied by higher Arg-1 and iNOS production. IL-17A expressed by TH17 cell increased obviously in PP and mLN, whereas IL-17F levels decreased in the presence of high levels of MDSC-derived Arg-1, proving once again that TH17 cell polarization is facilitated by Arg-1 expressed by MDSC during colitis (14, 21). γδT cells are another main source of IL-17A and IL-17F (57). We detected dramatic decreased percentage of γδT cells after quercetin treatment. It has been shown that E2 treatment decreases the percentage of IL-17A + γδT cells in joints of collagen-induced arthritis mice and inhibits IL-17 + γδT cell migration by up-regulating CCL20 after binding to ESR expressed by γδT cells (58). Quercetin might also reduce percentages of IL-17A + γδT cells after activating ESR expressed by γδT cells. Blocking IL-17A signals was a promising strategy to treat IL-17A related autoimmune diseases like psoriasis (59), rheumatoid arthritis (60) and ankylosing spondylitis (61, 62). However, anti-IL-17A treatment showed poor outcomes in IBD patients in a clinical trial (12). Importantly, in a double-blinded clinical trials, targeting IL-17A or IL-17R was ineffective to treat Crohn’s disease, rendering higher risk of adverse events, indicating a protective role of IL-17A in colitis studies (12, 13), which highlight the protective role of IL-17A in the IBD.

In conclusion, we have demonstrated distinct roles of IL-17A and IL-17F in DSS-induced colitis mice model which was dependent on increased Arg-1 secreted by MDSC after ESR/STAT3 activation. Our data highlight the role of Arg-1 in regulating IL-17A and IL-17F expression in the autoimmune response and provides further support for therapeutic approaches for IBD.

## Data Availability Statement

The raw data supporting the conclusions of this article will be made available by the authors, without undue reservation, to any qualified researcher.

## Ethics Statement

The animal study was reviewed and approved by the Subcommittee on Research Animal Care of the First Hospital of Jilin University.

## Author Contributions

ZM performed the experiments. ZM, YZ, and CH analyzed the data and wrote the manuscript. HY conceived the idea and supervised the project. All authors have read and approved the manuscript.

## Conflict of Interest

The authors declare that the research was conducted in the absence of any commercial or financial relationships that could be construed as a potential conflict of interest.
