# Supplementary material for: Myeloid-Derived Suppressor Cell-Derived Arginase-1 Oppositely Modulates IL-17A and IL-17F Through the ESR/STAT3 Pathway During Colitis in Mice
Source: Front Immunol. 2020 Apr 22;11:687. doi: 10.3389/fimmu.2020.00687 (PMC7188946; doi:10.3389/fimmu.2020.00687)
Supplement: Supplementary file 1 [file Data_Sheet_1.pdf]

Supplementary Material

1.1 Supplementary Figures 1

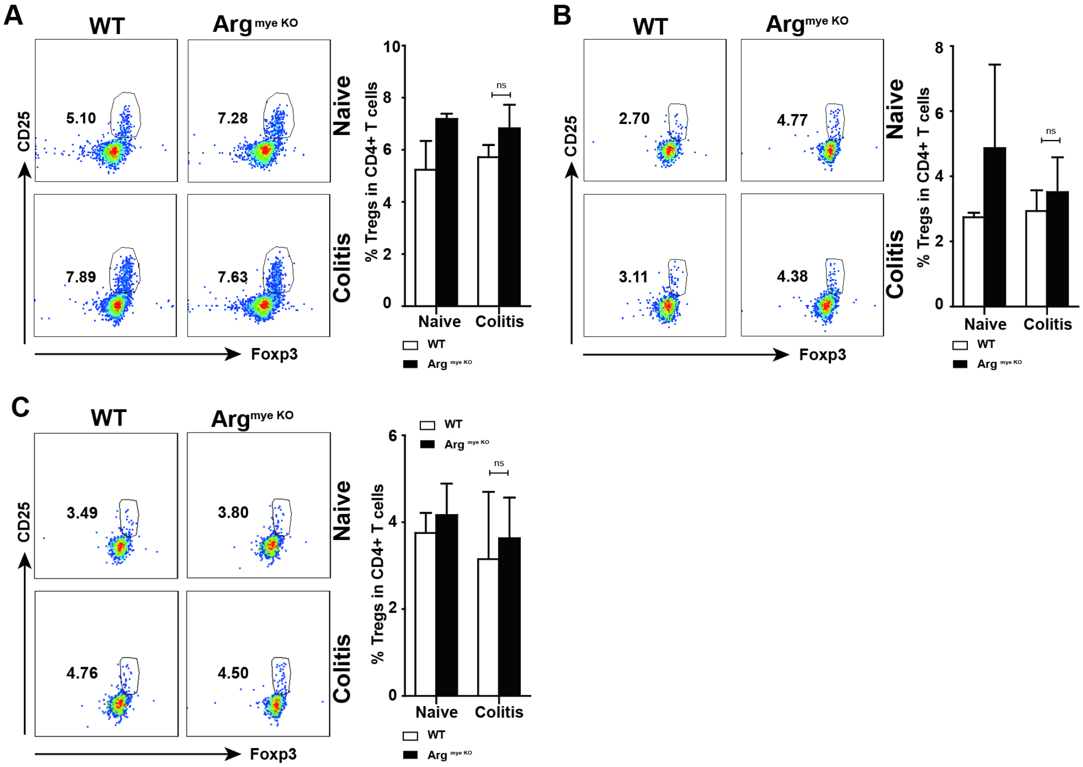

Supplementary Figure 1. Percentages of Tregs had no significant change between WT and Arg$^{mye\ KO}$ mice in DSS-induced colitis mice model.WT and Arg$^{mye\ KO}$ mice treated with 3.5% DSS for 9 days. Mice were sacrificed and PBMC, splenic cells and PP were isolated to detect Tregs as indicated. Flow cytometry showed the Tregs in PBMC (A), spleen (B) and PP (C). *P<0.05. Data are representative of three independent experiments with similar results. Quantification of signal was shown in bar graphs and error bars represent mean ± SD.

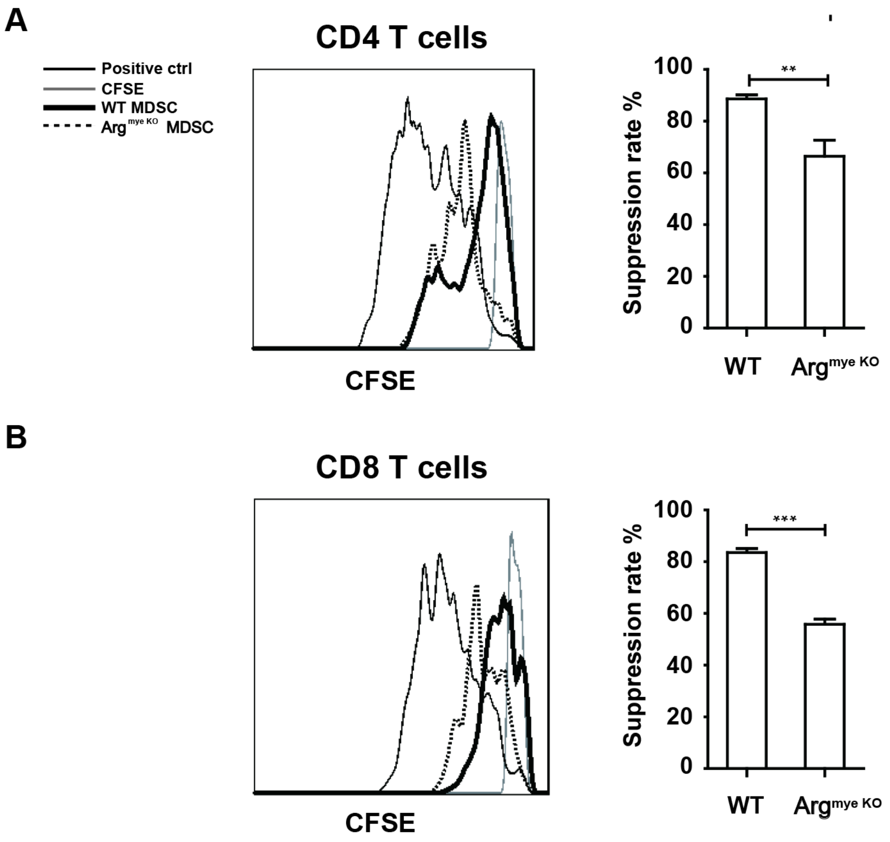

Supplementary Figure 2. Arg-1 deficiency impaired suppressive function of Arg$^{mye\ KO}$-derived MDSC. WT and Arg$^{mye\ KO}$ mice treated with 3.5% DSS for 9 days. Mice were sacrificed and splenic MDSC from WT and Arg$^{mye\ KO}$ mice were purified and co cultured with T cells as indicated. Flow cytometry showed the proliferation of CD4 (A) and CD8 (B) T cells co cultured with MDSC. **P< 0.01; ***P<0.001. Data are representative of three independent experiments with similar results. Quantification of signal was shown in bar graphs and error bars represent mean ± SD.

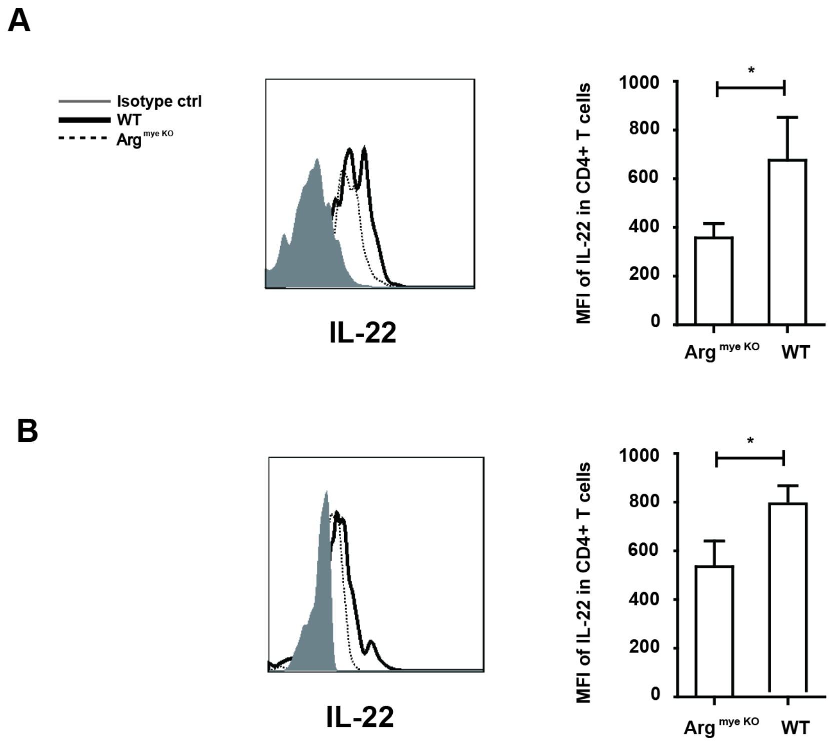

Supplementary Figure 3. Arg$^{\text{mye KO}}$ mice expressed lower level of IL-22 during colitis. WT and Arg$^{\text{mye KO}}$ mice treated with 3.5% DSS for 9 days. Mice were sacrificed and PP (A) and mLN (B) from WT and Arg$^{\text{mye KO}}$ mice were isolated to detect IL-22 level. gray, isotype; dotted, vehicle treatment; solid, quercetin treatment. *$P<0.05$. Data are representative of three independent experiments with similar results. Quantification of signal was shown in bar graphs and error bars represent mean ± SD.

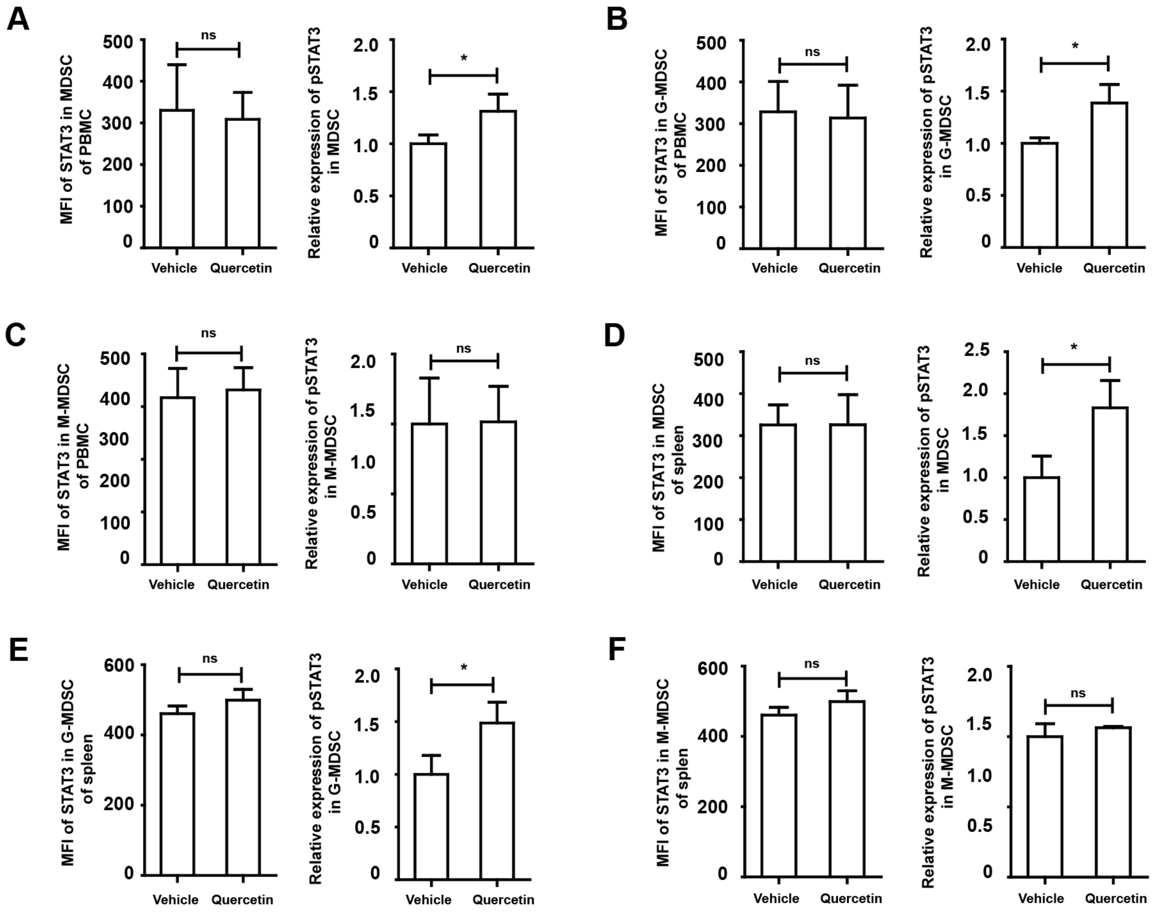

Supplementary Figure 4. Quercetin enhanced pSTAT3 level in MDSC during colitis. Colitic WT mice treated with quercetin and pSTAT3 level in MDSC (A) and subpopulations (B, C) of PBMC were detected. pSTAT3 level in splenic MDSC (D) and subpopulations (E, F) were detected. *P<0.05. Data are representative of three independent experiments with similar results. Quantification of signal was shown in bar graphs and error bars represent mean ± SD.

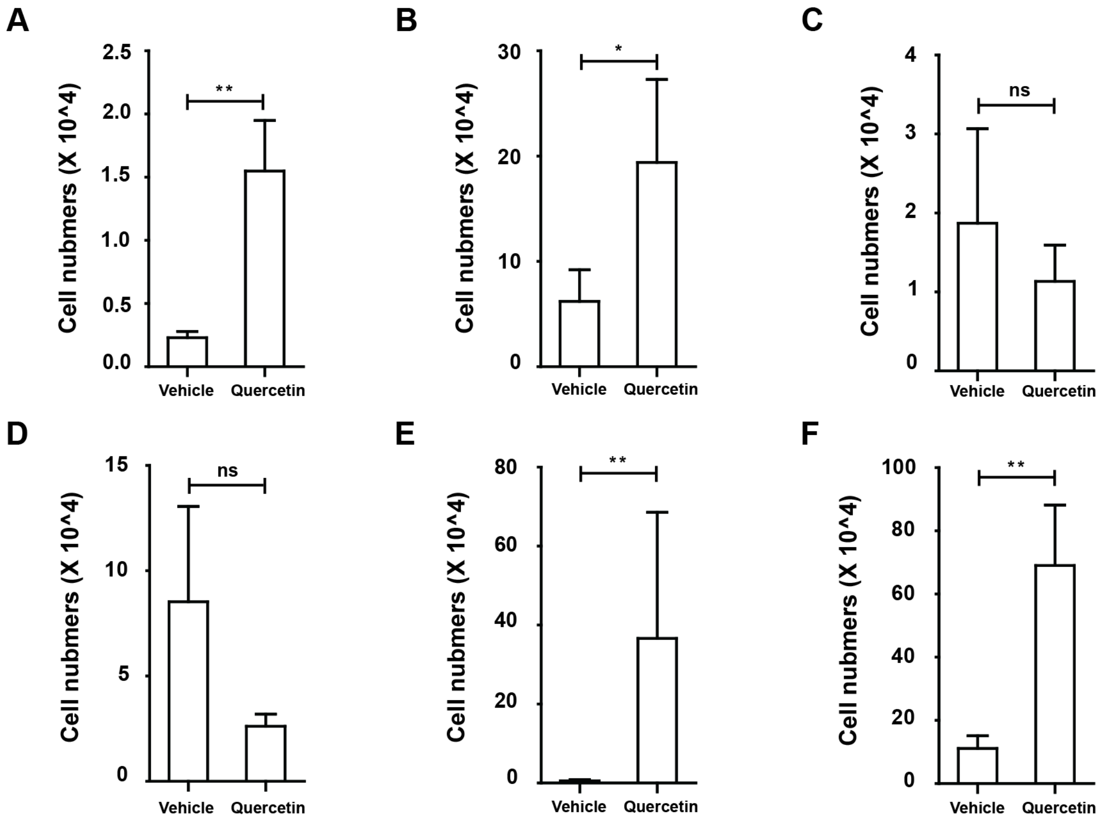

Supplementary Figure 5. Absolute counts of target cells in colitic mice. Colitic WT mice treated with quercetin and IL17A$^+$TH17 cells (A) and IL-17F$^+$TH17 cells (B) in PP or mLN (E, F) were detected. IL17A$^+$γδT cells (C) and IL-17F$^+$γδT cells (D) in PP were detected. *P<0.05; **P< 0.01. Data are representative of three independent experiments with similar results. Quantification of signal was shown in bar graphs and error bars represent mean ± SD.

A

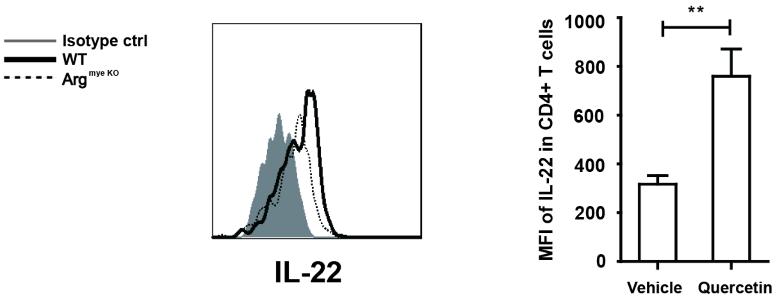

B

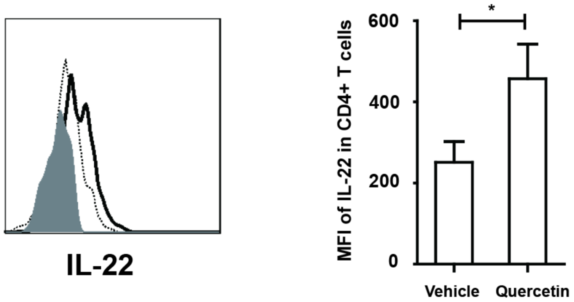

Supplementary Figure 6. Quercetin enhanced IL-22 level in mice during colitis. Colitic WT mice treated with quercetin and IL-22 level was detected in in PP (A) or mLN (B). gray, isotype; dotted, vehicle treatment; solid, quercetin treatment. *P<0.05. Data are representative of three independent experiments with similar results. Quantification of signal was shown in bar graphs and error bars represent mean ± SD.

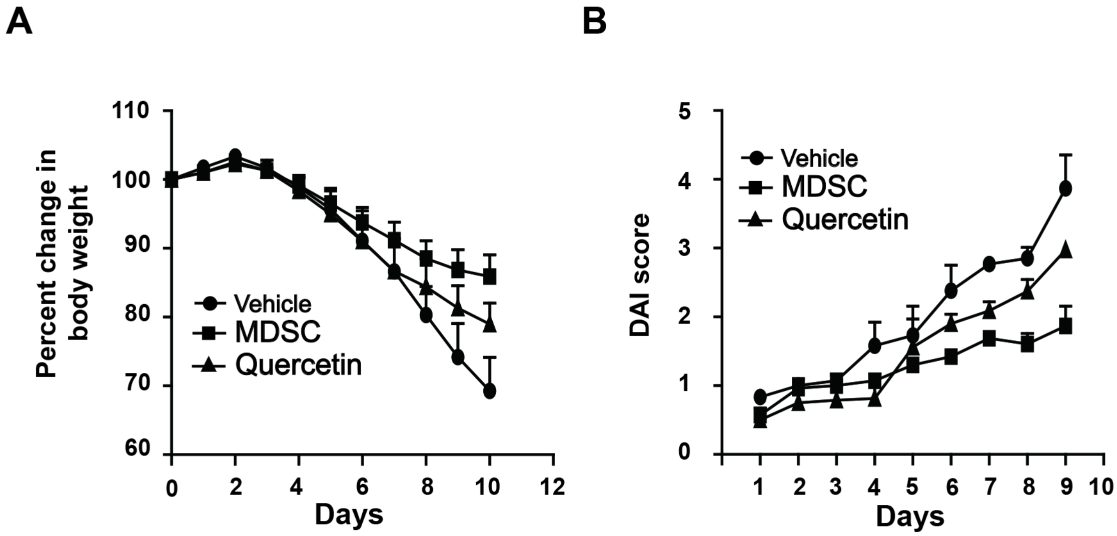

Supplementary Figure 7. Quercetin mildly alleviated colitis in Arg$^{mye\ KO}$ mice. Colitic Arg$^{mye\ KO}$ mice treated with quercetin or MDSC and monitored daily to record weight loss (A) and DAI score (B). Data are representative of three independent experiments with similar results. Quantification of signal was shown in bar graphs and error bars represent mean ± SD.
